# Progress in Surgery of the Intestines

**Published:** 1902-07-05

**Authors:** 


### Progress in Surgery of the Intestines.

**Intestinal Anastomosis.**—Frank[1] maintains that intestinal approximation is best effected by mechanical devices of the Murphy button type. The chief advantages of such devices are:—(1) Simplicity of application. (2) Saving of time. (3) Uniform coaptation of the peritoneal surfaces of the entire circumference of the approximated ends. (4) Prevention of bleeding at the seat of approximation. (5) A cicatrix that will not contract to a serious degree. (6) Exact juxtaposition of histological structures. (7) Minimising of risk of infection from the intestinal canal into the peritoneal cavity as no needle is used. There is also a reduction in mortality. With mechanical methods of the Murphy button type the mortality in gastro-enterostomy for non-malignant disease ranges from 2·5 to 14 per cent., in end-to-end union from 10·5 to 16 per cent.; and is only as high as 33 per cent. in malignant cases. With the suture methods the mortality ranges in gastro-enterostomy from 24·5 to 76·47 per cent., and in end-to-end approximation from 58 to 100 per cent.

**Extensive Resection of Small Intestine.**—Lauwers[2] thinks that it is possible without directly endangering life, to resect half of the small intestine, provided the operation involves a considerable portion or the whole of the ileum. The digestive capacity of the small intestine gradually diminishes in relation to the approach of this portion of the intestinal canal to the ileo-cæcal valve. Although removal of more than two metres of small intestine will probably be followed by gastro-intestinal disturbance, such functional troubles, the author states, have never caused death in such instances. He quotes a case in which he removed 36 inches of small intestine, and at the same time, by suturing the duodenal end of the divided small intestine to the sigmoid flexure, excluded 64 inches of the large intestine. Blayney[3] records a case of extensive resection for a crush of the intestine with extensive laceration of the mesentery in a boy aged 10 years. The intestine removed measured 8 feet 4½ inches in length. He gives a list of 33 cases in in which more than 1 metre (3 feet 3 inches) have been removed, and thinks that the teaching of clinical experience in adults would seem in cases uncomplicated by other lesions to point towards 200 cm. as being the limit about which the danger line fluctuates. In adults, when the intestine removed is less than this, intestinal disturbance will probably be absent, and when greater, present. Children must apparently be placed in a different category, for in a child, aged 8, 330 cm. (11 feet) have been removed without interference with nutrition or intestinal disturbance—possibly owing to a compensative hypertrophy of the intestine left behind.

**Unilateral Exclusion in Fæcal Fistula.**—The treatment of fæcal fistula, according to Delore and Patel,[4] practically resolves itself into one of two courses. Either the whole of the affected bowel is excised, or some form of anastomosis is undertaken with the object of throwing the loop out of action. Excision is to be recommended in but a small number of cases. The term "unilateral exclusion" should be confined to those cases alone in which the free current of intestinal contents in the diseased segment of bowel is entirely cut off, while yet a passage out exists for any fæcal or other contents left at the time of operation. The closing of the diseased segment at both ends would be an instance of bilateral exclusion. In five of the cases collected by the authors, the condition was one of fæcal fistula; the ileum was joined to some part of the large intestine with uniform success. They come to the following conclusions: 1 Unilateral exclusion belongs to the category of intestinal operations which, without directly attacking the seat of the lesion, cure by giving rest to this part. The operation has but a slight mortality, and is often very effective. 2. Applied to the cure of fæcal fistula, the results vary with the site of the lesion. (*a*) If the small intestine alone be affected, the result is exactly as follows a simple entero-anastomosis. The fæces accumulate in the lower segment, and the fistula persists. (*b*) In the case of the large intestine the results are often good. If the disease involves both ileum and cæcum, the ileum should be joined to the sigmoid. If the cæcum be free, then the ileum may be united to the ascending colon. 3. The suppression of the circulation of fæcal matter in the occluded segment is as successful under these latter circumstances as in bilateral exclusion. There seems no danger in tubercular lesions of the inoculation of healthy intestine below. 4. Unilateral exclusion is well adapted to the early stages of ileo-cæcal tubercle. Ulceration and fistula can by this means be prevented, and often the disease cured. The same authors regard enterectomy as the operation of choice for the cure of artificial anus. Maylard [5] and Bailing [6] write on the same subject.

**Jejunostomy.**—Cackovie [7] thinks that jejunostomy is an operation which presents no technical difficulties, and is indicated in the following conditions: (1) In cases of stenosis of the terminal portion of the œsophagus, in which, by reason of contraction of the stomach, gastrostomy is impracticable. (2) In cases of gastric carcinoma, in which a radical or a major palliative operation cannot be performed, or is found to be contra-indicated. (3) In cases of simple pyloric stricture and cicatricial contraction of the stomach, in which the patient is much exhausted or the stomach is extensively scarred and contracted. (4) In cases of gastric ulcer with frequent hæmorrhages, in which medicinal treatment fails to do any good, and death from inanition is threatened. (5) In cases of poisoning by caustic fluids likely to prove fatal through inanition or rupture of the stomach, and also for the results of tissue destruction caused by such poisons when no other operation is practicable. 6. In instances of insufficiency of suture after operations on the stomach, the results of which insufficiency cannot be removed in any other way. (7) Where a radical or a major palliative operation has had no immediate or remote success. Comparing the results of jejunostomy, and gastro-enterostomy, he finds that the mortality is about the same in both, whilst the duration of life in cases in which the patient has recovered from the immediate effects of surgical treatment is longer after the former operation.

**Carcinoma of the Colon.**—Walshaw [8] considers that the following symptoms should arouse our suspicions of carcinomatous stricture whilst still in the early stage: (1) Indefinite symptoms of abdominal disturbance, such as uneasiness, distension, eructations, constipation, symptoms called by the patient and often by his medical attendant indigestion, but not referable to the stomach and not benefited by ordinary routine treatment; (2) attacks of pain or spasm referred to the colon, often of daily occurrence and having no special connection with the time at which food is taken; (3) nearly always liquid stools with rarely a formed motion; (4) slowly progressive loss of weight; (5) constant desire to defecate.

[1] Brit. Med. Jour., March 8, 1902; Epitome, No. 157; and Annals of Surg., No. 1, 1902. [2] Brit. Med. Jour., Feb. 22, 1902, Epitome No. 120. [3] Brit. Med. Jour., Nov. 16, 1901, p. 1456. [4] Med. Chron., Aug. 1901, p. 362, and Rev. de Chir., 1901. [5] Med. Chron., April 1902, p. 1. [6] Birm. Med. Rev., June, 1901, p. 365. [7] Brit. Med. Jour., March 8, 1902, Epitome, No. 155. [8] Lancet, Aug. 17, 1901, p. 433.

